# Tacrolimus Prevents TWEAK-Induced PLA2R Expression in Cultured Human Podocytes

**DOI:** 10.3390/jcm9072178

**Published:** 2020-07-10

**Authors:** Leticia Cuarental, Lara Valiño-Rivas, Luis Mendonça, Moin Saleem, Sergio Mezzano, Ana Belen Sanz, Alberto Ortiz, Maria Dolores Sanchez-Niño

**Affiliations:** 1 IIS-Fundacion Jimenez Diaz, Universidad Autonoma de Madrid, Fundacion Renal Iñigo Alvarez de Toledo-IRSIN, 28040 Madrid, Spain; leticia.cuarental@quironsalud.es (L.C.); lara.valino@fjd.es (L.V.-R.); asanz@fjd.es (A.B.S.); 2 Red de Investigación Renal (REDINREN), Fundacion Jimenez Diaz, 28040 Madrid, Spain; 3 Nephrology Department, Centro Hospitalar Universitário São João, 4200-319 Porto, Portugal; luiscfmendonca@gmail.com; 4 Bristol Renal, University of Bristol, Bristol BS8 1TH, UK; cdmxs@bristol.ac.uk; 5 Laboratorio de Nefrologia, Facultad de Medicina, Universidad Austral de Chile, 5090000 Valdivia, Chile; mezzano.sergioa@gmail.com

**Keywords:** podocyte, nephrotic syndrome, membranous nephropathy, Fn14, TWEAK, tacrolimus, PLA2R

## Abstract

Primary membranous nephropathy is usually caused by antibodies against the podocyte antigen membrane M-type phospholipase A2 receptor (PLA2R). The treatment of membranous nephropathy is not fully satisfactory. The calcineurin inhibitor tacrolimus is used to treat membranous nephropathy, but recurrence upon drug withdrawal is common. TNF superfamily members are key mediators of kidney injury. We have now identified key TNF receptor superfamily members in podocytes and explored the regulation of PLA2R expression and the impact of tacrolimus. Data mining of single cell transcriptomics and glomerular transcriptomics data identified TNFRSF12a/Fn14 as the highest expressed TNF receptor superfamily gene in human membranous nephropathy, and this was confirmed by immunohistochemistry that also identified NFκB activation in membranous nephropathy podocytes. Additionally, glomerular transcriptomics identified PLA2R1 expression as being increased in membranous nephropathy in the parenteral administration of the Fn14 ligand TWEAK increased podocyte PLA2R expression in mice. Furthermore, in cultured human podocytes, TWEAK increased the expression of PLA2R as well as the expression of other genes recently identified by GWAS as linked to membranous nephropathy: NFKB1 and IRF4. Interestingly, IRF4 encodes the FK506-binding protein 52 (FKBP52), a protein associated with tacrolimus. Tacrolimus prevented the increased expression of PLA2R, NFKB1 and IRF4 induced by TWEAK in cultured podocytes. In conclusion, TWEAK upregulates the expression of PLA2R and of other genes linked to membranous nephropathy in podocytes, and this is prevented by tacrolimus. An impact of tacrolimus on the expression of PLA2R and other genes in podocytes may underlie its efficacy in treating the disease as well as the frequent recurrence of nephrotic syndrome upon tacrolimus withdrawal.

## 1. Introduction

Membranous nephropathy (MN) constitutes the main cause of nephrotic syndrome in nondiabetic white adults [1]. Its pathogenesis involves the formation of immune complexes in the subepithelial site of glomerular basement membrane, leading to podocyte damage [2]. MN may be associated with systemic diseases or exposures (i.e., secondary) but in most cases (80%), it is limited to the kidney (i.e., primary) [3]. If left untreated, around one third of patients with primary MN progress to end-stage renal disease [1]. The clinical approach to MN has changed since the recognition of its autoimmune pathogenesis and target antigens [4]. Most patients with primary MN have IgG4 circulating antibodies against the podocyte membrane M-type phospholipase A2 receptor (PLA2R), leading to subepithelial deposition of PLA2R-anti-PLA2R immune complexes [5,6]. PLA2R belongs to the mannose receptor family [7]. The role of PLA2R in podocyte biology is unclear, although it may be involved in binding or internalizing collagen through the fibronectin II domain [8]. Likewise, the mechanisms leading to the formation of autoantibodies are not well characterized [9]. A complex multi-hit process encompassing both genetic factors and environmental exposures has been proposed [10]. In this setting, the immune system may have an important role not only by stimulating the production of antibodies, but also by upregulating PLA2R expression [9]. Local complement activation by immunocomplexes ultimately leads to podocyte injury and proteinuria [11]. Patients with primary MN and persistent nephrotic syndrome are treated with immunosuppressor agents [1]. Currently used regimens include steroids, alkylating agents, calcineurin inhibitors and B cell depletion [6,12]. Calcineurin inhibitors are among the most commonly prescribed drugs worldwide for MN and achieve remission in 60–70% of patients [12]. Both cyclosporine and tacrolimus suppress T cells and T cell-dependent B-cell activation by blocking calcineurin phosphatase activity. However, recent data suggest that they might also directly protect podocytes through modulation of intracellular inflammatory pathways and stabilization of cytoskeleton [13]. Specifically, tacrolimus is one of the recommended calcineurin inhibitors for MN [14]. However, it is associated with a high rate of recurrence upon stopping therapy. Characterization of the factors regulating podocyte PLA2R expression could impact our understanding of disease pathogenesis and contribute to optimizing therapeutic strategies. In this regard, whether calcineurin inhibitors modulate podocyte PLA2R expression has not been explored so far.

We have now explored factors driving PLA2R expression in human podocytes and the impact of tacrolimus. Since TNF superfamily cytokines are key drivers of tissue injury and, specifically, of kidney injury [15], we used a data mining approach to identify potential regulators of PLA2R expression in podocytes by characterizing podocyte-expressed TNF receptor superfamily (TNFRSF) members. After identifying TNFRSF12a (Fn14) as one of the highest expressed TNFRSFs in podocytes in vivo and additionally as highly upregulated in glomeruli in human MN, we explored the impact of its ligand TWEAK on podocyte PLA2R expression in cultured cells and in vivo and demonstrated that TWEAK-induced podocyte PLA2R upregulation is prevented by tacrolimus. Additionally, TWEAK and tacrolimus modulated the expression of genes recently identified by genome-wide association study (GWAS) as being associated to MN [16].

## 2. Materials and Methods

### 2.1. Data Mining and Overall Experimental Design

**Figure 1:**
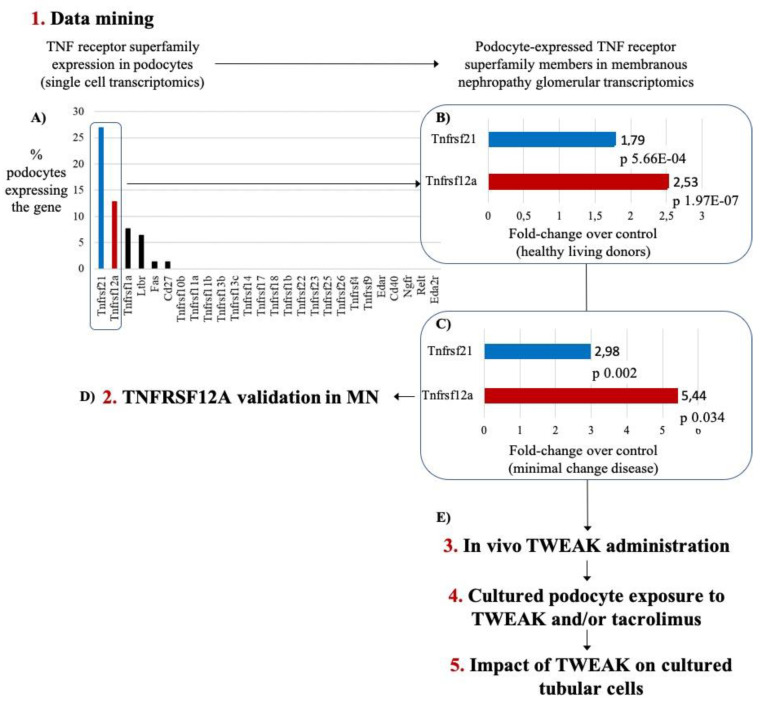
Experimental design and glomerular expression of TNF receptor superfamily (TNFRSF) members in human membranous nephropathy (MN). (**A**) Data mining (Step 1). Initially, the glomerular podocyte expression of TNFRSF family members was extracted from a publicly available murine kidney single cell transcriptomics dataset [17]. (**B**) Based on these results, *TNFRSF12a* (Fn14) and *TNFRSF21* were selected for further studies. Data on glomerular expression of these genes in MN (*n* = 21) vs. healthy living donors (*n* = 21) were extracted from Nephroseq [20] (http://v5.nephroseq.org/genesummarydetails?thresholds=pValue:0.05,rValue:0.5,foldChange:0&filters=89&geneIds=51330&geneSymbols=TNFRSF12A&selectedGene=51330; last accessed 19 March 2020). Both glomerular *TNFRSF21* and *TNFRSF12A* gene expression were significantly upregulated in MN, but only *TNFRSF12A* gene expression was increased more than two-fold. (**C**) Nephroseq 5.0 also identified a second dataset in which glomerular gene expression was compared for MN (*n* = 9) and minimal change nephrotic syndrome (*n* = 7) [21] (http://v5.nephroseq.org/genesummarydetails?thresholds=pValue:0.05,rValue:0.5,foldChange:1.5&filters=112&geneIds=27242,51330,22925&geneSymbols=TNFRSF21,TNFRSF12A,PLA2R1&selectedGene=51330; last accessed 17 April 2020). These results are in line with the observation in MN and healthy kidney donors. Glomerular transcriptomics data correspond to glomerular microarrays. (**D**) Increased expression of FN14, encoded by *TNFRSF12A*, was validated in human MN at the protein level (Step 2: Figure 2). (**E**) Once *TNFRSF12A* was identified as a gene of interest, its potential functional impact was addressed. For that, first, the ligand (TWEAK) for the receptor (Fn14) encoded by *TNFRSF12A* was administered systemically to mice and kidney immunostaining for PLA2R was performed (Step 3: Figure 3). Once it was shown that TWEAK indeed upregulated PLA2R expression in vivo in podocytes, we addressed in cultured human podocytes whether TWEAK upregulated PLA2R and other genes related to the pathogenesis of MN and the impact of tacrolimus (Step 4: Figure 4). Finally, to provide context, proximal tubular cell responses were assessed (Step 5).

In order to identify potential regulators of PLA2R expression in podocytes among members of the TNFRSF, we explored recently available single cell transcriptomics databases [17] to identify TNFRSF members expressed in podocytes, as well as the Nephroseq v 5.0 online database (http://v5.nephroseq.org/) to search for glomerular transcriptomics data that had explored TNFRSF expression in human MN (Figure 1). Once TNFRSF12a/Fn14 was identified among the highest expressed TNFRSF genes in podocytes in vivo and the most upregulated in glomeruli in human MN, the impact of its ligand TWEAK on podocyte PLA2R expression was explored in mice and in cultured human podocytes and the impact of tacrolimus on TWEAK-induced PLA2R expression was explored in cultured human podocytes. Additionally, we queried a previously published in-house database that had evaluated by transcriptomics arrays the impact of 100 ng/mL TWEAK on gene expression in murine cultured proximal tubular epithelial MCT cells [18,19]. Specifically, we queried this database for the impact of TWEAK on the expression of genes identified by GWAS to be associated with MN [16].

Finally, whether *PLA2R1*, the gene encoding PLA2R, is differentially expressed in human MN was addressed by searching Nephroseq for datasets containing glomerular data for human MN and controls.

### 2.2. Animal Models

All animal work was conducted according to national and international guidelines and was approved by the Fundacion Instituto Investigacion Sanitaria Fundacion Jimenez Diaz animal research ethics committee (PROEX 036/16). Euthanasia was performed by cervical dislocation. Female 12 to 14-week-old C57/BL6 mice from the IIS-Fundacion Jimenez Diaz animal facilities were administered 0.75 μg TWEAK or saline vehicle intraperitoneally and were euthanized 24 h after injection (*n* = 5 per group). The dose of TWEAK was calculated on the basis of cell culture dose-response experiments for an extracellular volume of 7.5 mL/mouse and was previously shown to elicit biological responses in the kidneys in vivo [22,23,24,25,26,27]. Kidneys were perfused in vivo with ice-cold saline and processed for immunohistochemistry.

### 2.3. Cells and Reagents

Human podocytes were a gift from Prof Moin Saleem (University of Bristol). This is an immortalized cell line transfected with a temperature-sensitive SV40 gene construct and a gene encoding the catalytic domain of human telomerase [28,29,30]. At a permissive temperature of 33 °C, cells remained in an undifferentiated proliferative state and divide. Raising the temperature to 37 °C resulted in growth arrest and differentiation to the parental podocyte phenotype. Undifferentiated podocyte cultures were maintained at 33 °C in RPMI 1640 medium with penicillin, streptomycin, ITS (insulin, transferrin, selenite) and 10% fetal calf serum. Once cells reached 70–80% confluence, they were fully differentiated by culture at 37 °C for at least 14 days [28,29]. Cells were cultured in serum-free medium 24 h prior to the addition of stimuli and throughout the experiment.

Recombinant human soluble TWEAK (Millipore, Billerica, MA, USA) was used at 100 ng/mL, based on prior dose-response experiments [24,26,31]. Tacrolimus (USBiological) stock solutions (10 mg/mL) was dissolved in ethanol for a final concentration of 25 ng/mL. This concentration is within the clinical significant range, since recommended blood trough levels range from 5 to 20 ng/mL early post-transplant and from 5 to 15 ng/mL for maintenance therapy (https://www.ema.europa.eu/en/documents/referral/prograf-article-30-referral-annex-i-ii-iii_en.pdf; accessed 17 April 2020). Tacrolimus or vehicle were added one hour prior to stimulation with TWEAK.

### 2.4. Western Blot

Cell samples were homogenized in lysis buffer then separated by 10% or 12% SDS-PAGE under reducing conditions and transferred to PVDF membranes (Millipore, Bedford, MA, USA), blocked with 5% skimmed milk in PBS/0.5% *v*/*v* Tween 20 for 1 h, washed with PBS/Tween, and incubated with mouse monoclonal anti-PLA2R (1:1000, Abcam, Cambridge, UK). Blots were washed with PBS/Tween and subsequently incubated with appropriate horseradish peroxidase-conjugated secondary antibody (1:2000, Amersham, Aylesbury, UK). After washing, blots were developed with the chemiluminescence method (ECL, Amersham, UK), and then probed with the mouse monoclonal anti-α-tubulin antibody (1:5000, Sigma, St, Louis, MO, USA) [32]. Levels of expression were corrected for minor differences in loading.

### 2.5. Quantitative Reverse Transcription-Polymerase Chain Reaction

One microgram of RNA was isolated using Trizol (Invitrogen, Paisley, UK) and reverse-transcribed with the High Capacity cDNA Archive Kit. Real-time PCR was performed on a ABI Prism 7500 PCR system with pre-developed primer and probe assays (Applied Biosystems, Foster City, CA, USA) using the DeltaDelta Ct method. Expression levels are given as ratios to GAPDH [18].

### 2.6. Immunohistochemistry

Immunohistochemistry in murine samples was performed as previously described on paraffin-embedded 3 µm tissue sections [33]. The primary antibody was mouse monoclonal anti-PLA2R (1:1000, Abcam, Cambridge, UK) and anti-WT1 (Dako, Glostrup, Denmark) in the same section or in specular images. Negative controls included incubation with a non-specific immunoglobulin of the same isotype as the primary antibody. Sections were counterstained with Carazzi’s hematoxylin.

Immunohistochemistry in human samples was performed in leftover tissue after percutaneous renal biopsy and complete diagnostic evaluation for nephrotic syndrome at the Division of Nephrology (Austral University, Valdivia, Chile). Control human kidney specimens (*n* = 5) were taken from normal portions of renal tissue from patients who underwent surgery because of localized renal tumors. Study samples were MN specimens (*n* = 13, aged 53 ± 8 years, 6 females, serum creatinine 1.3 ± 0.7 mg/dL, proteinuria 5.4 ± 3.0 g/d). Immunohistochemistry was carried out in paraffin-embedded tissue sections 5 μm thick [34]. The primary antibody was rabbit anti-Fn14 (Cell Signaling Technology, Danvers, MA, USA). Sections were counterstained with Carazzi’s hematoxylin. Negative controls included incubation with a non-specific immunoglobulin of the same isotype as the primary antibody. The local ethics committee approved the study protocol and informed consent was obtained (PIC 24/2016). This was a reanalysis of previously processed samples [31].

### 2.7. Southwestern Histochemistry

Southwestern histochemistry was performed in human MN and control kidney samples. The probe was synthetic sense DNA 5′-AGTTGAGGGGACTTTCCCAGGC-3′ containing a consensus sequence for NF-κB (GIBCO BRL, Life Technology, Gaithersburg, MD, USA) [34]. After annealing with their complementary DNA (80 °C for 2 min), each probe was labeled with digoxigenin (DIG oligonucleotide 3-end labeling kit; Boehringer Mannheim, Mannheim, Germany). The following as negative controls were used: (1) no probe; (2) mutant digoxigenin-labeled NF-κB probe (sense 5′-AGTTGAGGCTCCTTTCCCAGGC-3′) at the same concentration as the original probe; and (3) competition with a 200-fold excess of unlabeled NF-κB, followed by incubation with labeled probe.

### 2.8. Statistics

Statistical analysis was performed using SPSS 11.0 statistical software. The results are expressed as mean ± SEM. Significance at the *p* < 0.05 level was assessed by Student’s *t* test for two groups of data and ANOVA for three or more groups with Bonferroni post hoc.

## 3. Results

### 3.1. Fn14 (Tnfrsf12a) Is among the Top TNFRSF Members Expressed by Podocytes In Vivo

The TNF superfamily of cytokines includes key contributors to kidney injury such as TNF, Fas ligand, TRAIL, TWEAK and others [15,35,36]. In order to identify potential TNF superfamily members of interest for MN, we used a data mining approach of publicly available databases. First, we searched for TNFRSF members expressed by podocytes. In murine kidney single cell transcriptomics studies, *Tnfrsf21*, *Tnfrsf12a*, *Tnfrsf1a*, *Ltbr*, *Fas* and *Cd27* were the only TNFRSF members expressed in more than 1% of podocytes (Figure 1A) [17]. Of podocyte-expressed TNF superfamily members, only *Tnfrsf21* and *Tnfrsf12a* were expressed in more than 10% of podocytes. For *Tnfrsf12a*, these results are consistent with prior immunohistochemistry studies [31]. Thus, *Tnfrsf21* and *Tnfrsf12a* were selected for further studies.

### 3.2. Glomerular Fn14 Is Upregulated in Human MN

We then searched Nephroseq 5.0 for TNFRSF members expressed under baseline conditions in podocytes and overexpressed in human MN. The database contained information from a manuscript that compared glomeruli from patients with MN versus healthy living donors [20]. Both glomerular *TNFRSF21* and *TNFRSF12A* gene expression were significantly upregulated in MN, but only *TNFRSF12A* gene expression was increased more than two-fold (Figure 1B). Nephroseq 5.0 contained information from a second manuscript that had compared MN to minimal change nephrotic syndrome [21]. Again, both glomerular *TNFRSF21* and *TNFRSF12A* gene expressions were significantly upregulated in MN, but, again, the fold-change was higher for *TNFRSF12A* gene expression (Figure 1C). Since glomerular *TNFRSF12A* encodes the Fn14 receptor for TWEAK, while *TNFRSF21* encodes an orphan receptor (DR6), and given the higher differential glomerular expression of *TNFRSF12A*, we decided to explore the impact of TWEAK on PLA2R expression.

Additionally, Nephroseq identified two datasets which had data on glomerular *PLA2R1* expression in human MN. Glomerular PLA2R expression was increased in 21 glomerular MN samples as compared to 151 samples from patients with other kidney diseases, including 25 focal segmental glomerulosclerosis and 14 with minimal change disease (Fold Change: 1.559, p: 9.43e-5), as well as in 9 patients with MN compared with 15 patients with focal segmental glomerulosclerosis [15] (Fold Change: 1.737, p: 0.019) or 3 patients with other causes of nephrotic syndrome (Fold Change: 1.532, p: 0.024) [20,21] (http://v5.nephroseq.org/genesummarydetails?thresholds=pValue:0.05,rValue:0.5,foldChange:1.5&filters=89,90&geneIds=22925&geneSymbols=PLA2R1&selectedGene=22925; last accessed 16 June 2020).

**Figure 2:**
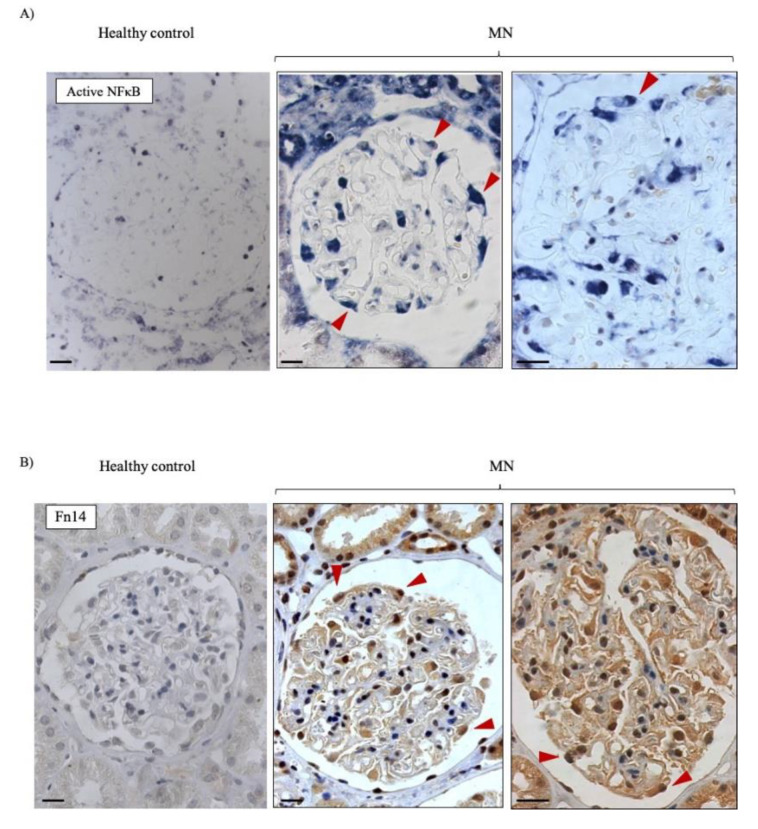
NFκB activation and Fn14 expression in human MN and human control kidney. (**A**) NFκB Southwestern (original magnification ×40 and ×60) and (**B**) Fn14 immunohistochemistry (Original magnification ×40 and ×60). Arrowheads point to stained cells sitting outside the capillaries and protruding towards Bowman’s capsule, consistent with podocytes. Scale bar: 20 µm. Supplementary Figure S1 shows additional controls.

In human MN, immunohistochemistry disclosed increased Fn14 expression in podocytes from all 13 specimens tested (Figure 2), while little Fn14 staining was observed in control glomeruli. Additionally, Southwestern histochemistry disclosed NFκB activation in cells in the periphery of MN glomeruli that had podocyte morphology (Figure 2). This was not observed in control specimens.

### 3.3. TWEAK Increases PLA2R Expression in Podocytes In Vivo

**Figure 3:**
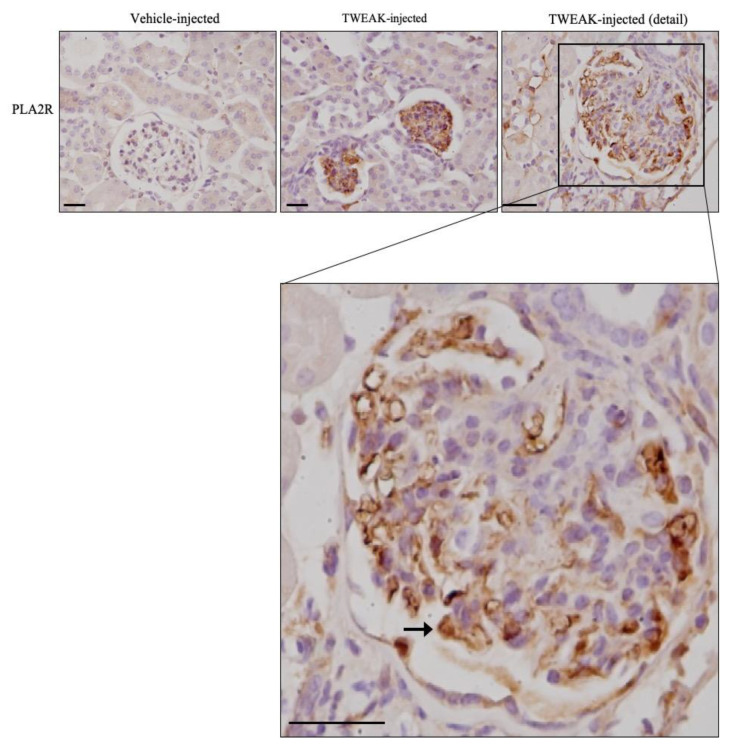
TWEAK increases PLA2R expression in podocytes in vivo. Immunohistochemistry showing that systemic TWEAK injection in healthy mice increased glomerular PLA2R immunostaining in vivo after 24 h. Under baseline control conditions, glomerular PLA2R immunostaining was negative, as expected from single cell transcriptomics studies that only reflected low (6.5% of the cells) *Pla2r* gene expression in endothelial cells and lack of expression in all other kidney cells [17]. The pattern of glomerular PLA2R immunostaining in TWEAK-injected mice, in the periphery of glomerular capillary walls and in the body of cells sitting outside the capillaries and protruding towards Bowman’s capsule was consistent with the increased podocyte expression of PLA2R (arrow). Representative images from *n* = 4 animals per group. Original magnification ×20, scale bar 20 µm.

Systemic TWEAK administration has long been known to promote an inflammatory response in the kidneys [22,26]. Additionally, TWEAK administration increased PLA2R expression in the glomeruli of TWEAK-injected mice (Figure 3). The pattern of immunostaining in the periphery of glomerular capillary walls and in the body of cells sitting outside the capillaries and protruding towards Bowman´s capsule was consistent with increased podocyte expression of PLA2R (Figure 3, detail). Co-staining with anti-PLA2R and the marker of podocytes anti-WT1-1, or the staging of adjacent specular sections with anti-PLA2R on one section and anti-WT-1 on the adjacent section further localized glomerular PLA2R expression to podocytes (Supplementary Figure S3).

### 3.4. TWEAK Increases PLA2R Expression in Cultured Human Podocytes and This Is Inhibited by Tacrolimus

**Figure 4:**
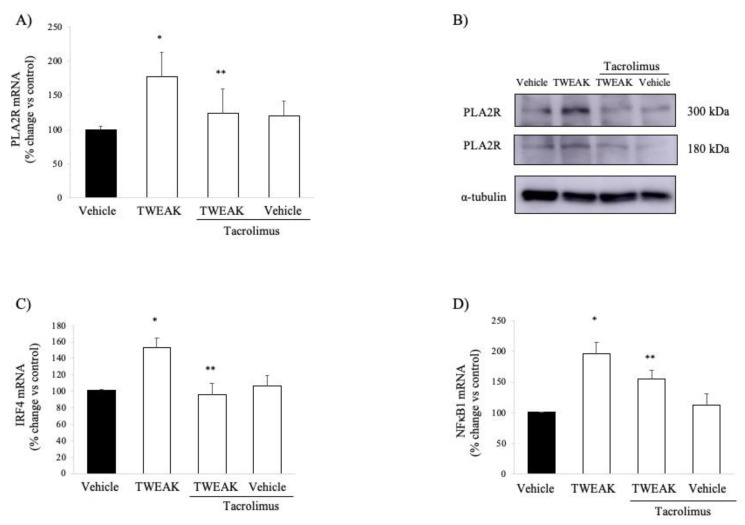
TWEAK increases PLA2R expression in cultured human podocytes: impact of tacrolimus. (**A**,**B**) TWEAK 100 ng/mL increases PLA2R mRNA (**A**) and protein (**B**) expression in cultured human podocytes after 6 h. This was prevented by pretreatment with 25 ng/mL tacrolimus. Real time RT-PCR and representative Western blot. Two main bands were detected in Western blot, likely representing the full-length and extracellular domain of this glycosylated protein that may be generated by alternative splicing or proteolytic processing. Mean ± SD of three independent experiments. * *p* < 0.05 vs. Vehicle, ** *p* < 0.05 vs. TWEAK alone. Complete gel shown in Supplementary Figure S2. (**C**,**D**) TWEAK also increased the mRNA levels of *IRF4* (**C**) and *NFKB1* (**D**) assessed by quantitative RT-PCR. This was also prevented by tacrolimus. * *p* < 0.02 vs. vehicle; ** *p* < 0.02 vs. TWEAK alone.

Next, we studied the impact of TWEAK on PLA2R expression in cultured human podocytes. TWEAK increased PLA2R expression at the mRNA and protein levels within 6 h (Figure 4A,B).

Interestingly, loci associated to human MN in GWAS studies included *PLA2R* and also *NFKB1* and *IRF4*, in addition to certain human leukocyte antigen (HLA) genes [16]. In this regard, TWEAK also increased *IRF4* mRNA (Figure 4C) and *NFKB1* mRNA (Figure 4D) levels in cultured podocytes. Thus, TWEAK modulated the expression of all three non-HLA genes that have been linked to human MN by GWAS studies.

This effect of TWEAK was shared by podocytes and cultured tubular cells for *NFKB1*, according a transcriptomics analysis (fold-increase TWEAK 100 ng/mL vs. vehicle in proximal tubular cells: 1.36 at 6 h, *p* = 0.0047) [18,19] (Supplementary Table S1). However, TWEAK did not increase the mRNA encoding PLA2R (Fold increase TWEAK vs. control conditions 1.05 at 6 h, *p* = 0.65) or IRF4 (Fold increase TWEAK vs. control conditions 1.05 at 6 h, *p* = 0.41) in cultured tubular cells (Supplementary Table S1).

TWEAK-induced upregulation of PLA2R expression in human podocytes was abrogated by clinically relevant concentrations of tacrolimus (Figure 4A,B). Tacrolimus also prevented TWEAK-induced *NFKB1* upregulation (Figure 4C). IRF-4 associates with the FK506-binding protein 52 (FKBP52) [37] and it is a key target of tacrolimus, being downregulated by tacrolimus and cyclosporine [38,39]. In this regard, tacrolimus also prevented TWEAK-induced *IRF4* upregulation (Figure 4D).

## 4. Discussion

The main findings are that the TNFRSF member TNFRSF12a/Fn14, a receptor for TWEAK upregulated in glomeruli from patients with MN, is likely to contribute to the pathogenesis of MN through the increased expression of PLA2R. Indeed, TWEAK increased PLA2R in vivo in murine podocytes as well as in cultured human podocytes. TWEAK additionally upregulated the expression of the only two other non-HLA genes that have been ever linked by GWAS to MN: *NFKB1* and *IRF4*. Interestingly, TWEAK-induced changes in podocyte gene expression were prevented by tacrolimus, suggesting a further potential mechanism of action of tacrolimus in MN.

For the first time, we identified TWEAK as a driver of PLA2R overexpression in podocytes under inflammatory conditions. In this regard, we expand on the general deleterious effect that TWEAK has on podocytes. Thus, TWEAK triggers NFκB activation and secretion of chemokines that may attract immune cells [31]. However, these effects are non-specific from the point of view of the type of glomerular injury in which they may be pathogenic. We now expand TWEAK actions on podocytes to MN-specific pathogenic mechanisms; that is, upregulation of the target antigen of MN autoimmunity.

In addition to *PLA2R1* and ancestry-specific HLA alleles (DRB1*1501 in East Asians, DQA1*0501 in Europeans, and DRB1*0301 in both ethnicities), a recent GWAS for primary MN in 3782 cases and 9038 controls of East Asian and European ancestries identified two previously unreported loci, *NFKB1* (rs230540, OR = 1.25, *p* = 3.4 × 10^−12^) and *IRF4* (rs9405192, OR = 1.29, *p* = 1.4 × 10^−14^) as associated to MN [16]. Together with *PLA2R1* and the HLA alleles, these GWAS loci explained 32% of disease risk in East Asians and 25% in Europeans [16]. Interestingly, TWEAK also increased the expression of *NFKB1* and *IRF4* mRNA in cultured podocytes [31]. Thus, TWEAK and/or Fn14 could be therapeutic targets in MN. In this regard, neutralizing anti-TWEAK antibodies have already been tested in clinical trials for lupus nephritis (NCT01930890). Since current immunosuppressive therapy has a lag time for complete remission, dependent on the mechanism of action (e.g., autoantibody half-life for rituximab) [6,40], adjuvant nephroprotective therapy that protects podocytes during this lag time in which the immune response is suppressed and the beneficial impact is felt could be helpful.

In addition to identifying the TWEAK/Fn14 axis as a driver of MN-related gene expression in podocytes, we identified tacrolimus as a suppressor of this TWEAK-driven response. Tacrolimus is currently recommended as one of the alternatives to treat primary MN by KDIGO [14] and is undergoing clinical trials to further define its role in therapy, given the high recurrence rate after stopping the drug [41]. This high recurrence rate is not observed for other immune suppressive approaches and may be related to a local action of tacrolimus on podocytes. In this regard, our findings of tacrolimus limiting an acute response to inflammatory cytokines that drives MN-related gene expression, including PLA2R availability, is compatible with the disappearance of protection once tacrolimus is stopped.

While tacrolimus prevented the TWEAK-induced increase in *PLA2R1*-mRNA, it did not decrease the low-level constitutive *PLA2R1* expression in podocytes. Thus, it could be argued that in patients with MN, tacrolimus may not have an effect if there is no trigger that increases *PLA2R1* expression such as TWEAK. In this regard, TWEAK is present in the circulation, and the activity of the system is mainly regulated by the availability of its receptor Fn14 [36]. In this regard, the expression of *TNFSRF12A*, the gene encoding Fn14, is increased in human MN, suggesting overactivity of the TWEAK/FN14 axis in human MN. It may additionally be argued that PLA2R may not be actually upregulated in human MN and that the increased immunostaining observed is due to the presence of the protein in immune depots. While acknowledging the difficulty of studying gene expression in human glomeruli form MN patients and the fact that biopsies may be performed at different stages of the disease, two different transcriptomics datasets identified *PLA2R1* as being overexpressed in human MN, supporting a potential role of tacrolimus in decreasing antigen availability in human MN. In any case, any extrapolation to potential clinical events from our experimental data should be cautious.

A number of limitations should be acknowledged. Thus, tacrolimus effects were only tested in cultured human podocytes and the impact of tacrolimus on TWEAK-driven PLA2R expression was not tested in vivo. In this regard, there is no easy way, beyond repeat renal biopsy, to test the impact of tacrolimus on human podocytes in vivo in patients with MN who are started on tacrolimus. Among the strengths, we have taken a non-biased approach that was validated in cultured cells, an animal model and in human MN and have explored the impact of TWEAK/Fn14 on clinically relevant molecules that were very recently identified by GWAS, as well as the impact of clinically relevant tacrolimus concentrations on TWEAK modulation of PLA2R expression and expression of GWAS identified genes in human podocytes. However, a further limitation to be addressed is the issue of the potential nephrotoxicity of tacrolimus. Thus, further studies should address whether tacrolimus still inhibits the effect of TWEAK at concentrations under 5 ng/mL in vivo.

**Figure 5:**
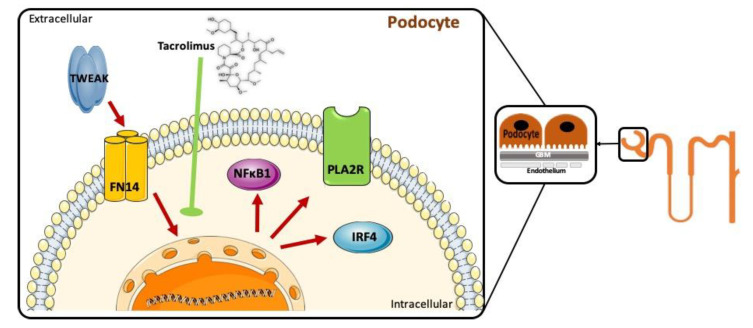
TWEAK activation of Fn14 results in increased podocyte expression of non-human leukocyte antigen (HLA) genes associated by GWAS to human membranous nephropathy. Fn14 is the receptor for the inflammatory cytokine TWEAK. The activity of the TWEAK/Fn14 axis is usually regulated by receptor availability, although TWEAK levels may also increase in the course of inflammation. After identifying upregulation of *TNFRSF12A*, the gene encoding Fn14, and of *PLA2R1* in human membranous nephropathy, we have shown that TWEAK increases PLA2R expression in human podocytes, thus potentially contributing to increased antigen availability and the generation of local immune complexes. Tacrolimus decreased the TWEAK-induced expression of PLA2R and other genes associated to membranous nephropathy by GWAS. This may contribute to its therapeutic impact in the clinic.

In conclusion, our findings contribute to a better understanding of MN pathogenesis and therapy. Thus, we have identified an inflammatory cytokine TWEAK, that through the activation of the Fn14 receptor upregulates in podocytes all three non-HLA genes (*PLA2R, NFKB1, IRF4*) associated with MN in human GWAS studies (Figure 5). TWEAK itself may become a therapeutic target. Additionally, by identifying a potential mechanism of action of tacrolimus directly on podocytes that may contribute to the characteristic induction of remission plus high recurrence rate, our findings may help to optimize therapy for MN.

## Supplementary Materials

The following are available online at https://www.mdpi.com/2077-0383/9/7/2178/s1, Figure S1. Fn14 immunostaining controls in human tissue; Figure S2: Full gel for figure 4.B, Figure S3: PLA2R immunostaining in TWEAK injected mice; Table S1: Cultured tubular cell transcriptomics data. MCT cells were stimulated for 6 h with vehicle or TWEAK 100 ng/mL.
