# Supplementary material for: Tacrolimus Prevents TWEAK-Induced PLA2R Expression in Cultured Human Podocytes
Source: J Clin Med. 2020 Jul 10;9(7):2178. doi: 10.3390/jcm9072178 (PMC7408934; doi:10.3390/jcm9072178)
Supplement: Supplementary file 1 [file jcm-09-02178-s001.pdf]

Journal of
Clinical Medicine

MDPI

Table S1. Cultured tubular cell transcriptomics data. MCT cells were stimulated for 6 hours with vehicle or TWEAK 100 ng/mL.

Gene Symbol	Control 1	Control 2	Control 3	TWEAK 1	TWEAK 2	TWEAK 3	TWEAK/Control ratio for experiment 1	TWEAK/Control ratio for experiment 2	TWEAK/Control ratio for experiment 3	TWEAK/Control ratio (all)	p-value	Gene Accession
Nfkb1	10,15	9,95	9,76	10,50	10,33	10,39	1,27	1,31	1,54	**1,36**	0,0047	NM_008689
Pla2r1	5,67	5,36	5,08	5,38	5,31	5,65	0,82	0,96	1,49	**1,05**	0,6487	NM_008867
Irf4	4,38	4,24	4,32	4,48	4,29	4,38	1,07	1,03	1,05	**1,05**	0,4134	NM_013674

Journal of
Clinical Medicine

MDPI

Figure S1. Fn14 immunostaining controls in human tissue. A) Controls for human immunostaining: no primary antibody, minimal change disease, healthy control. Scale bar 20 μm. B) Controls for human immunostaining: immune-complex mediated glomerulonephritis. Scale bar 20 μm.

A

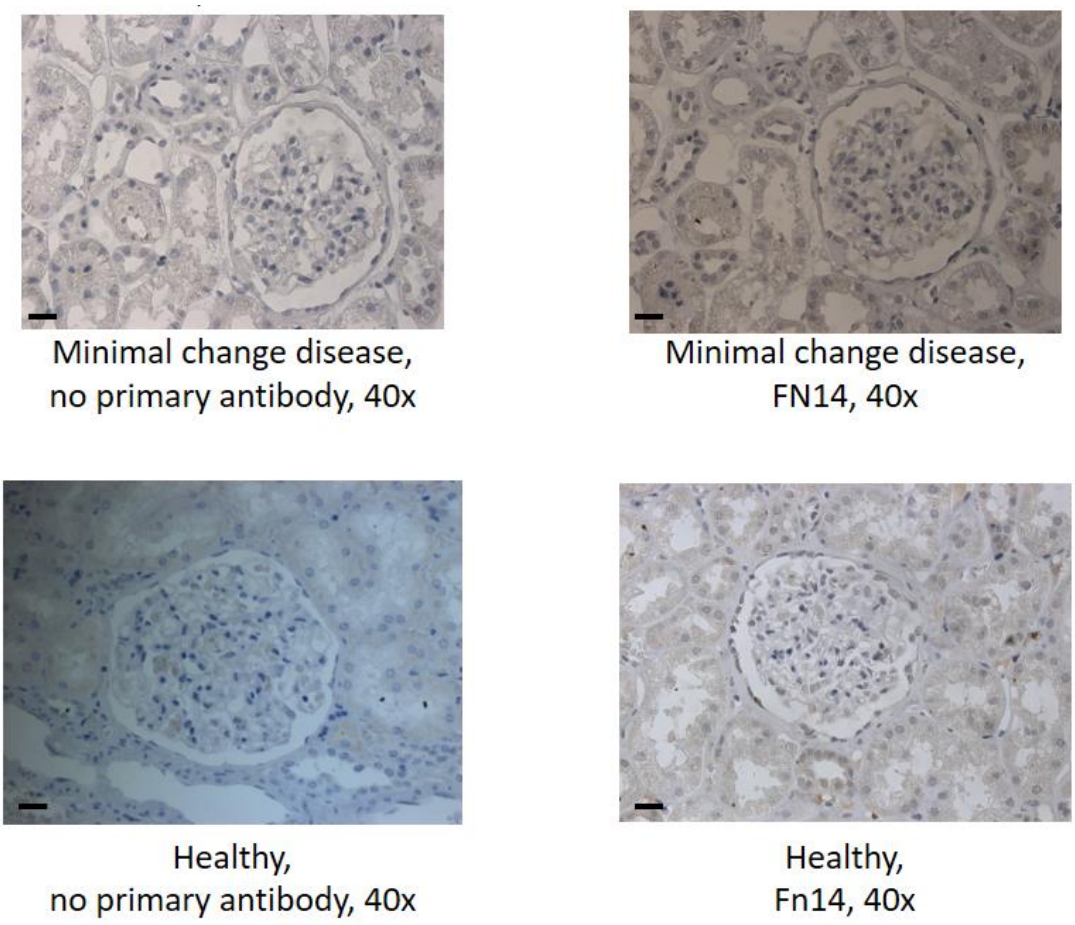

B

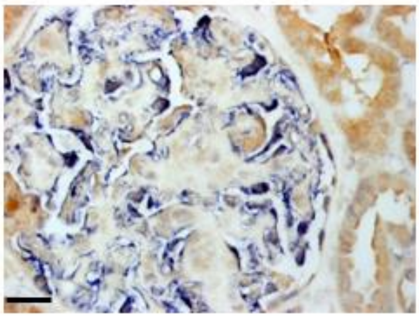

Sistemic lupus erythematous
WT1, Fn14, 60x

Journal of Clinical Medicine

MDPI

Figure S2. Full gel for figure 4.B.

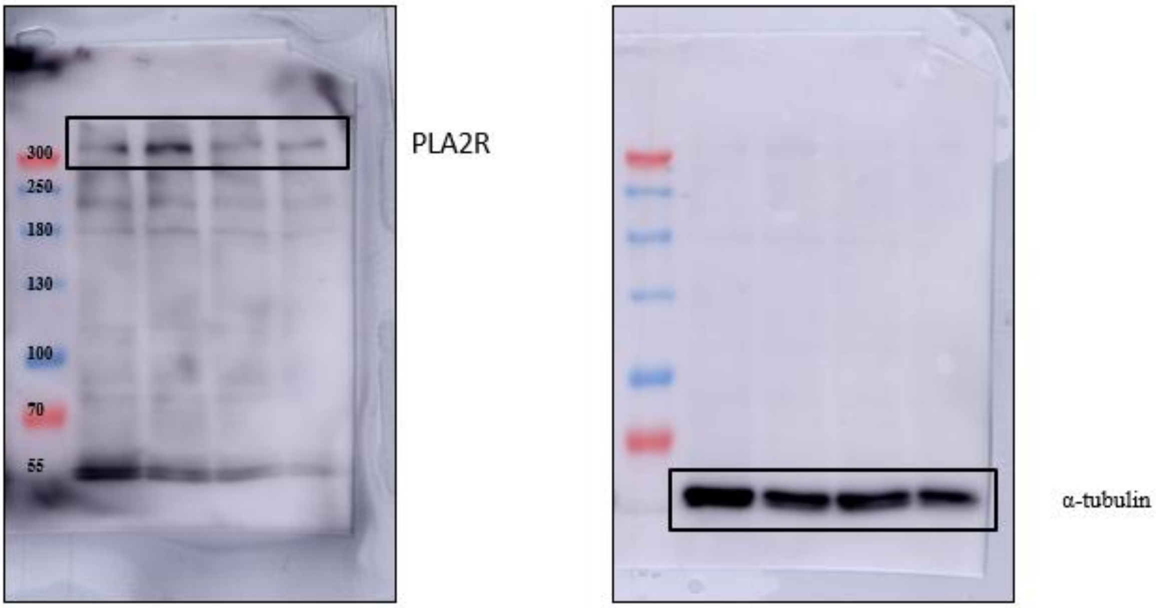

Journal of
Clinical Medicine

Figure S3. PLA2R immunostaining in TWEAK injected mice. A) Panoramic view of sections of TWEAK-injected mice stained for the podocyte marker WT1 or for PLA2R. Scale bar 20 µm. B) Specular images of adjacent sections of TWEAK-injected mice kidneys stained for the podocyte marker WT1 or for PLA2R. Arrows point podocytes. Scale bar 20 µm. C) Co-staining for WT1 and for PLA2R in TWEAK-injected mice kidneys. Arrows point podocytes. Scale bar 20 µm. D) Negative control. TWEAK-injected mice kidney was staineded as for anti-PLA2R but omitting the primary antibody.

A

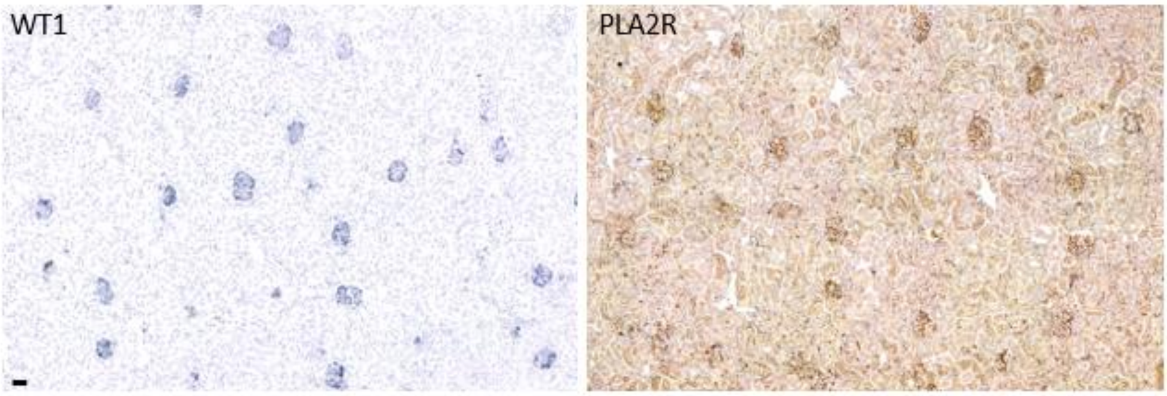

B

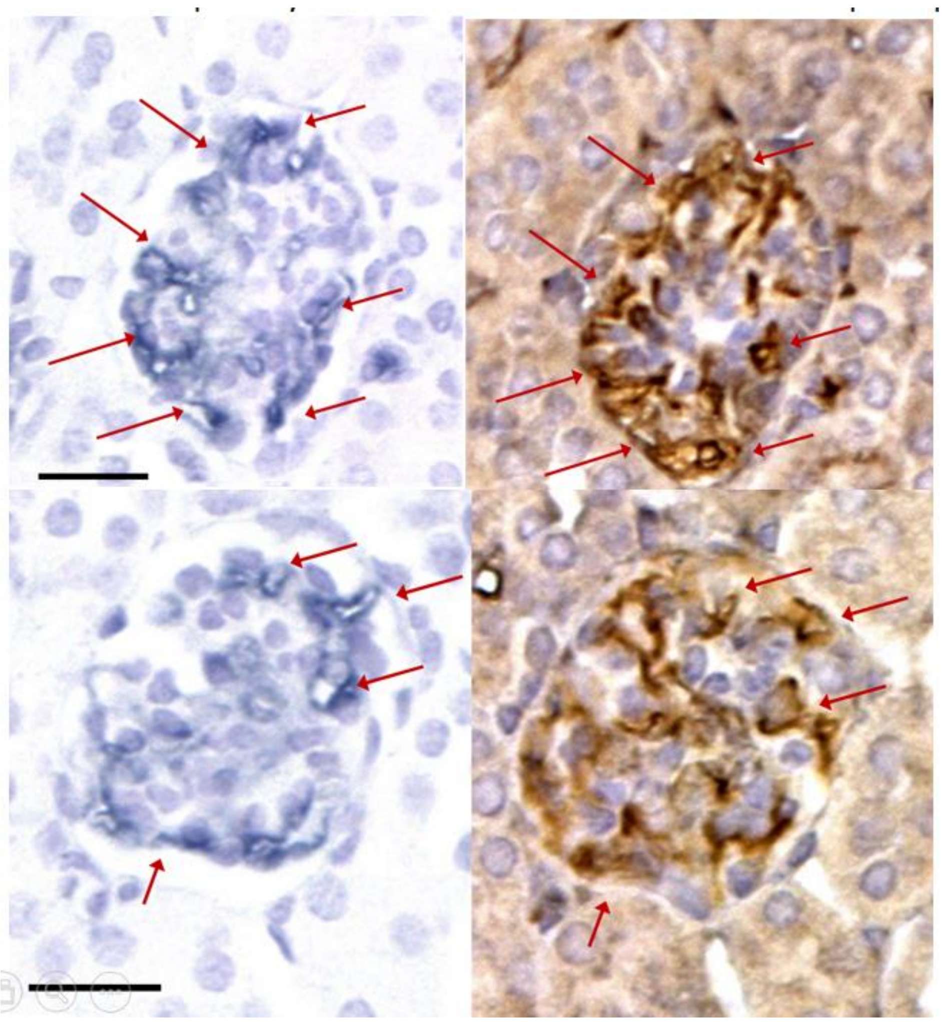

C

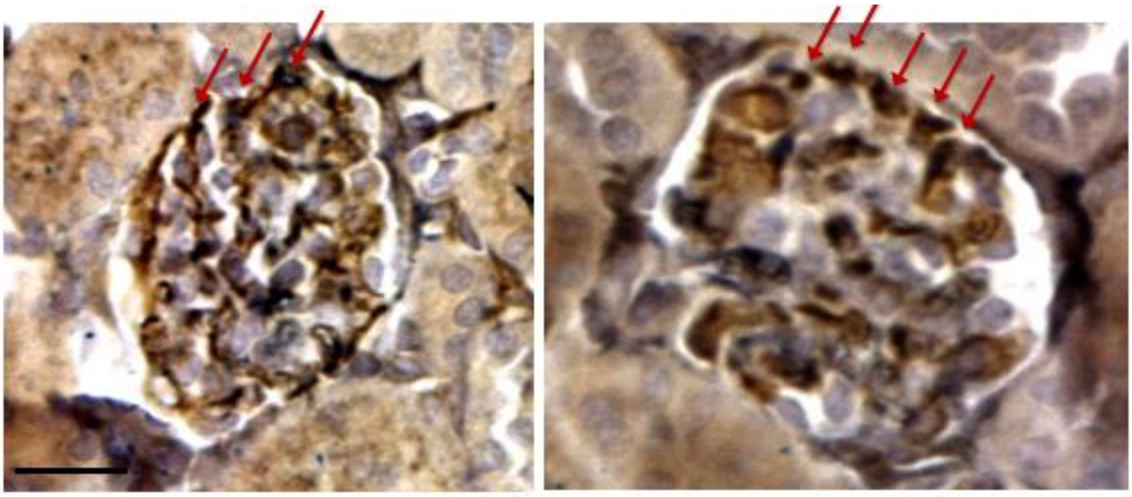

D

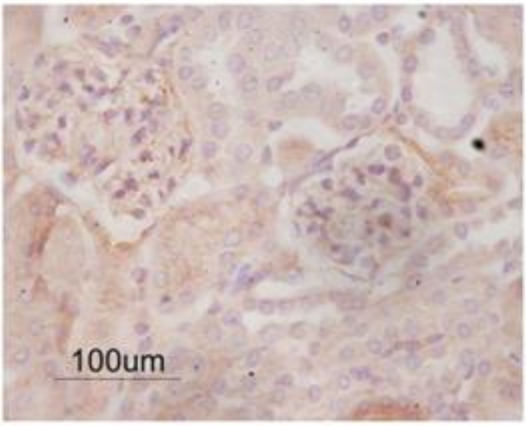
100um
